# Voltage-Gated Sodium Channel Dysfunctions in Neurological Disorders

**DOI:** 10.3390/life13051191

**Published:** 2023-05-16

**Authors:** Raffaella Barbieri, Mario Nizzari, Ilaria Zanardi, Michael Pusch, Paola Gavazzo

**Affiliations:** Institute of Biophysics, Via de Marini 6, 16149 Genova, Italy; raffaella.barbieri@ibf.cnr.it (R.B.); mario.nizzari@ibf.cnr.it (M.N.); ilaria.zanardi@ibf.cnr.it (I.Z.); michael.pusch@ibf.cnr.it (M.P.)

**Keywords:** Nav channel blockers, epilepsy, FHM3, intellectual disability, neurodegeneration, Alzheimer’s disease, Parkinson’s disease, amyotrophic lateral sclerosis

## Abstract

The pore-forming subunits (α subunits) of voltage-gated sodium channels (VGSC) are encoded in humans by a family of nine highly conserved genes. Among them, *SCN1A*, *SCN2A*, *SCN3A*, and *SCN8A* are primarily expressed in the central nervous system. The encoded proteins Nav1.1, Nav1.2, Nav1.3, and Nav1.6, respectively, are important players in the initiation and propagation of action potentials and in turn of the neural network activity. In the context of neurological diseases, mutations in the genes encoding Nav1.1, 1.2, 1.3 and 1.6 are responsible for many forms of genetic epilepsy and for Nav1.1 also of hemiplegic migraine. Several pharmacological therapeutic approaches targeting these channels are used or are under study. Mutations of genes encoding VGSCs are also involved in autism and in different types of even severe intellectual disability (ID). It is conceivable that in these conditions their dysfunction could indirectly cause a certain level of neurodegenerative processes; however, so far, these mechanisms have not been deeply investigated. Conversely, VGSCs seem to have a modulatory role in the most common neurodegenerative diseases such as Alzheimer’s, where SCN8A expression has been shown to be negatively correlated with disease severity.

## 1. Introduction

One in six people worldwide present a neurological condition. Age remains the biggest risk factor for developing a neural disease; thus this frequency is going to increase since the life spans in many countries continue to extend.

The topic of this review concerns the involvement of Nav channel isoforms in some inherited, genetic or non-genetic neurological or neurodegenerative pathologies affecting the Central Nervous System (CNS). A description of the molecular mechanisms underlying some common neural diseases is outlined and contextualized in light of impairments of the Nav channels.

The research activity on neural diseases, such as it is currently intended, began several decades ago, and for this reason, a large amount of information has been accumulated. However, over time widely divergent data have been published on specific points, probably due to the level of complexity of the topic. Throughout the paper, we will focus on some of these puzzles trying to provide the univocal and shared explanation that finally emerged from the scientific community itself.

### VGSC Molecular Architecture, Function and Expression Patterns

The family of voltage-gated sodium channels (VGSC) includes nine isoforms (Nav1.1–Nav1.9) encoded in humans from nine different genes (*SCNA1-5*, *SCNA7-10*). They are composed of the principal alpha-subunit, which is essential and sufficient for channel functioning and can be eventually associated with the accessories ß-subunits (ß1–ß4) which regulate gating, kinetics and channel surface density. 

**Figure 1:**
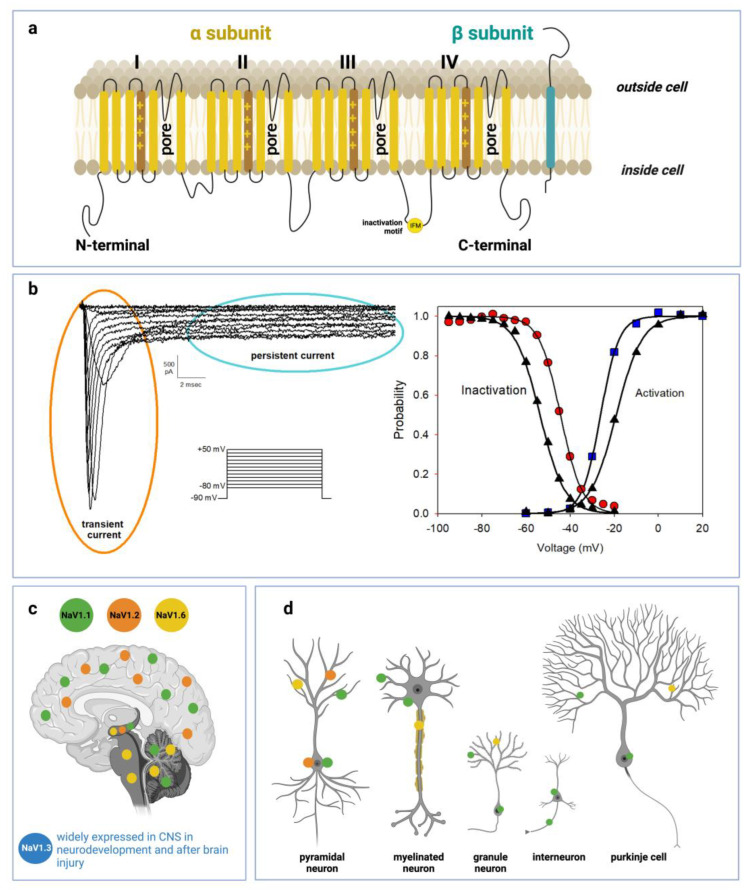
Voltage-gated sodium channel structure, function and distribution in CNS: (**a**) Schematic representations of α-subunit and auxiliary β-subunit of Nav channels. The α-subunit of the channel consists of four homologous domains (DI–DIV) made of six transmembrane helices (S1–S6). The voltage-sensor is localized in the fourth helix (S4) in each domain. The loops between S5 and S6 in each domain form the pore. (**b**) Left side: representative current traces recorded from a HEK293 cell transiently transfected with a cDNA encoding Q1489H/Nav1.1 mutant channel. Current traces were evoked from a series of 20 ms depolarizing pulses from −80 to +50 mV in 10 mV increments, starting from a holding potential of −90 mV. Q1489H mutation exhibits a large persistent current. Right side: curves of voltage dependence of steady-state activation (blu squares) or inactivation (red circles) are represented. The same curves are represented also for a *WT-SCN1A* transfected cell (black triangles) not reported in the figure. Lines stand for the Boltzmann function fits (**c**) Distribution of Nav1.1, Nav1.2, Nav1.3, and Nav1.6 in human brain regions. (**d**) Cellular and subcellular distribution of Nav1.1, Nav1.2, Nav1.3, and Nav1.6 in human brain.

The nine subtypes of the main α-subunit share a high degree of homology with more than 50% conserved sequences in the transmembrane and extracellular domain [1]. α-subunits are about 2000 amino acid residues long, have a molecular weight of 260 kDa and are organized in one long polypeptide chain folded in four linked internally repeated homologous domains (DI-IV), each containing six transmembrane segments (S1–S6), a pore region formed by the loop between S5 and S6 helices and a voltage sensor located at the level of the S4 segment containing several positively charged residues (see Figure 1). 

Accessories ß subunits are 33–36 kDa transmembrane proteins harboring a single transmembrane region. They are associated with the main subunit by non-covalent interactions or disulfide bonds [2] and interact with cell adhesion molecules or intracellular matrix proteins such as ankyrin [3,4] thus contributing to cell migration and adhesion [5].

Among the nine VGSC human isoforms, Nav1.1, 1.2, 1.3 and 1.6 are the primary Nav channels expressed in the CNS, whereas Nav1.7, 1.8 and 1.9 are mostly restricted to the peripheral nervous system (PNS); Nav1.4 is expressed in skeletal muscles and Nav1.5 in the cardiac muscle [1,6]. Among the prevalent channels in central neurons, Nav1.1 and Nav1.3, encoded by the *SCNA1* and *SCNA3* genes respectively, are preferentially expressed in the cell body [7] while Nav1.2 is more concentrated in unmyelinated axons and dendrites [7] and Nav1.6 in myelinated axons and dendrites [8]. 

Besides the unique distribution along the different neuronal compartments, CNS Nav also exhibits a well-defined expression pattern in a variety of cortical neuron subtypes with specific roles in excitability. Even if broadly detectable in CNS, Nav1.1 is predominant in inhibitory Gamma Aminobutyric Acid-ergic (GABAergic) interneurons [9,10], while Nav1.2 prevails in excitatory neurons [11] and Glutamatergic neurons [12]. Similarly, Nav1.6 is expressed in neocortical excitatory neurons [13]. Nav1.3 is expressed in CNS only during embryonic and neonatal life, while during infancy its expression declines and is progressively replaced by other isoforms, especially Nav1.1 and 1.2 [14]. 

Voltage-gated sodium channels are determinants for the initiation, propagation and regulation of action potentials in neuronal circuits. Their conductance is strictly influenced by the transmembrane potential to the change of which they respond extremely quickly in the order of a few milliseconds. At resting potential, in the closed non-conductive state, their ion flux is prevented by the intracellular activation gate, located where the four S6 helices meet. Conversely, when transmembrane potential shifts to less negative values, an outward dislocation of the S4 helix occurs and, being this domain physically coupled to the pore region through the S4–S5 linkers, triggers pore opening and initiation of ion flux [15] (see Figure 1). S4 movement also initiates the process of fast inactivation, provoking the dislocation of the intracellular loop linking DIII and DIV, resulting in the occlusion of the pore. Nav channels also undergo another type of inactivation, named slow inactivation, provoked by prolonged or repetitive stimulation and connected with a movement of S6 in DIII with S1 in the DIV domain which induces pore collapse [16].

The channel activity is enhanced and a gain of function (GoF) phenotype is observed when, due to a mutation in the gene sequence or to the administration of a compound:

- the voltage of current activation shifts toward more negative values;
- inactivation shifts toward more positive values;
- the current persists longer;
- the recovery from inactivation is faster;
- the current density, i.e., the number of functioning channels expressed per membrane unit area increases

On the contrary, a loss of function phenotype (LoF) develops when current activation occurs at less negative voltages and inactivation at less positive, or when the current density decreases or recovery from inactivation is slower. 

Since the studies on the squid giant axon in the fifties [17] VGSCs have historically been among the first ion channels to have been hypothesized and then identified. However, a number of decades before, at the beginning of the twentieth century, the history of chemistry-driven ion channel drug discovery had its beginning with the identification of VGSC modulators such as lidocaine used as local anesthetics or anticonvulsants such as phenytoin [18] which are still in clinical use. Over time, VGSCs have been discovered to be targeted by many natural toxins and therapeutic drugs. This pharmacological interest has positively stimulated the search for structure determination at the atomic level of Nav channels or portions of them. This has been fundamental in making therapeutic drugs readily available to challenge the multiple diseases in which these channels are involved. Currently a well-documented classification of seven different drug toxin binding sites (site 1 to 7) and a local anesthetic binding site have been defined [19,20]. The drugs in use for the treatment of neurological and neurodegenerative diseases in which VGSCs are differently implicated will be described in the next sections of this review.

## 2. Methods

The information reported in this review was collected by interrogating Public Databases, mainly PUBMED and Scopus, combining the specific keywords of the review, as well as Nav channel, epilepsy, migraine, intellectual disability, and neurodegenerative disease. 

Among the large amounts of recovered results, we chose to highlight the information reported in seminal papers and the most relevant recent findings which have the advantage of leveraging all prior knowledge. 

## 3. VGSC Associated Neurological Disorders: Channelopathies

Mutations in VGSC isoforms cause diseases called channelopathies. Among the others, VGSCs have primary importance in genetic neurological disorders such as epilepsy and migraine. In the context of this topic, their functional impairments are widely investigated and pharmacological treatments have been defined or are under study. Epilepsy is one of the most common neurological disorders characterized by recurrent seizures that can be also associated with cognitive, psychological and social problems [21]. In the wide spectrum of the hundreds of epileptogenic genes, *SCN1A* and *2A* are the most relevant, but also *SCN3A* and *8A* have been found to be correlated with forms of epilepsy. However, even if the knowledge of the roles that Nav1.1, Nav1.2, and Nav1.6 channels play in epilepsy has increased greatly in the past decade, the prediction of the clinical outcome of a variant in any of these channels remains first unknown [22].

### 3.1. Nav1.1 Associated Epilepsies: Dravet Syndrome and GEFS+ 

Nav1.1 is widely expressed in the CNS and is by far the most frequent target of epileptogenic mutations which lead to several syndromes exhibiting a wide range of severity. At the moment about 1500 pathogenic mutations of *SCN1A* have been described [23] and the majority of them, over 900, are associated with the Severe Myoclonic Epilepsy of Infancy (SMEI), a rare and grave form of epilepsy described for the first time by Charlotte Dravet in 1978 renamed Dravet syndrome in 1989 [24]. This disease, an autosomal dominant disorder, displays its symptoms already in the first year of life with seizures often associated with high body temperatures, and worsens during the second year, when failure of motor coordination and cognitive impairments emerge. The syndrome in the majority of cases is associated with de novo mutations determining frame shifts or premature termination sequences in one copy of *SCN1A*, resulting in non-functional Nav1.1 channels and pathogenic haploinsufficiency [25]. As a consequence, Dravet patients fail to produce a sufficient level of functional channel and undergo a number of impairments actually correlated with a Nav1.1 LoF effect; but how is this decrease of Nav1.1 activity, which would predict reduced excitability in the brain, correlated with the occurrence of epileptic seizures, thus with an increase in excitability? An explanation of this apparent paradox was first proposed by Catterall’s group [9]. A SMEI mouse model was generated through the ablation of Scn1a gene, and the heterozygous phenotype *Scn1a+/−* was assumed to mimic human SMEI. Currents recorded from hippocampal neurons showed a remarkable reduction of the Nav current in inhibitory GABAergic neurons of heterozygous and null homozygous animals with respect to wild type. However, the same reduction was not observed in glutamatergic excitatory pyramidal cells, thus indicating that Nav1.1 is predominant in GABAergic neurons. Accordingly loss-of function mutations of Nav1.1 results in a reduction of the brain inhibitory excitability determining an imbalance of brain excitation over inhibition which is at the basis of the epileptic seizure. This study besides being crucial for the advancement in the knowledge of the basic mechanisms underlying Dravet syndrome and many other forms of epilepsy as well, raises in the meanwhile the question of the need to design different therapies in relation to the Nav1.1 epileptogenic affected gene.

The above results have been overall confirmed in numerous subsequent studies performed on other mouse models and also on patients. However, a complex scenario is emerging in which, besides the damage induced from the presence of the mutation and of the subsequent seizures, compensatory remodeling mechanisms may take place depending on neuron type, genetic background and other factors, thus adding new variables to the genotype-phenotype correlation and to the clinical outcome of epilepsy and probably of other neurological diseases [26]. Additionally, generalized epilepsy with febrile seizure plus (GEFS+) syndrome is related to about 50 mutations of the Nav1.1 channel. GEFS+ is a milder form of epilepsy not associated with cognitive impairments and with symptoms usually well controlled by antiepileptic drugs. The confusing picture that emerged from initial studies of functional effects of GEFS mutations in transfected cells or *Xenopus laevis* oocytes [27,28] was later unambiguously clarified from in vivo study on a transgenic mouse model expressing the Scn1a GEFS+ mutation R1648H, consistent with the idea that R1648Q mutation led to a reduction in interneuron excitability [29] not associated with cognitive impairments. Several other milder forms of febrile seizures mostly in children have been also associated with Nav1.1 mutations even though it has been supposed that further precipitating factors, such as single nucleotide polymorphisms may contribute to the severity of the disease [30]. 

### 3.2. Nav1.1 Associated Migraine: Familial Hemiplegic Migraine Type 3 (FHM3) 

Genetic analyses of a group of dominant monogenetic diseases called familiar hemiplegic migraine (FHM), led to the identification of specific migraine genes [31]. Three genes have been recognized as causative agents for FHM1, 2, and 3 respectively, all of which are involved in membrane ion transport. FHM3 is caused by mutations in *SCN1A* [32,33] and constitutes a severe subtype of migraine with aura, characterized by some degrees of hemiparesis, sometimes associated with other neurological symptoms, such as epilepsy or blindness.

Several aspects of FHM3 have long been incompletely understood. Even the question of whether pathological mutations (12 known so far) lead to loss or to gain of function was not resolved. Recently using novel Knock-In (KI) FHM3 mouse models and heterologous expression of FHM3 mutations, several studies have contributed to uncovering the molecular and cellular mechanisms underlying FHM3. Results in vitro are in line with a major gain of function effect as a possible explanation of familial hemiplegic migraine 3; indeed a shift of the steady state inactivation to more positive voltages, an accelerated recovery from inactivation, and an increase of the persistent current were observed in all tested FHM3 mutation (L1649Q, L1670W, F1774S, Q1489H, I1498M, F1499L, M1500V, F1661L) [34,35]. 

Accordingly, in the KI L1649Q-FHM3 mouse model, hyperactivity of the Nav1.1 channel, which is predominantly expressed in inhibitory GABAergic interneurons was observed resulting in hyperexcitability of interneurons; this element contributes to lower the threshold to elicit the onset of the Cortical Spreading Depression (CSD), a neural pathological mechanism consisting in a wave of neuronal depolarization slowly propagating across cortex and strictly linked to migraine aura [36]. The combination of these investigations shed light on the molecular defects causing FHM3 that can be potentially relevant also for other non-genetic forms of migraine with and without aura with which FHM3 may share molecular pathogenic mechanisms.

### 3.3. Other Epileptogenic Nav Isoforms

Nav1.2 is mainly expressed in excitatory neurons of the cortex and hippocampus and its mutant variants are mostly related to the benign familial neonatal infantile seizures (BFNIS), infantile West syndromes, Early infantile epileptic encephalopathy (EIEE), epilepsy of infancy with migrating focal seizures (EIMFS) [37]. About 100 Nav1.2 epileptogenic mutations have been identified so far with several hot spots recognized mostly at the level of the selectivity filter, the pore, the voltage sensor and of N- and C-termini [12,38]. Overall there is no consensus on the molecular process causing the forms of epilepsy associated with Nav1.2 since in vitro functional studies have demonstrated that LoF or GoF mutations can be involved in the epileptic phenotype. Several cases have been shown in which Na1.2-related seizures were not controlled by antiepileptic drugs (AED).

The first epileptogenic mutation of Nav1.3, K345Q, was identified in 2010 and associated with a case of partial cryptogenic epilepsy [39,40].

Since then different hereditary or de novo mutations of Nav1.3 have been identified related to epilepsy, generally with a GoF phenotype. Interestingly several features such as epileptic encephalopathy and polymicrogyria, have been associated exclusively with Nav1.3-related epilepsies and have not been reported in other channelopathies [41]. However, so far the clinical data on Nav1.3-associated epilepsies are quite scarce and accordingly efficacious therapeutic treatments still need to be optimized. 

It is worth mentioning that in the last decades, a pivotal role of Nav1.3 have emerged in nervous system injury and neuropathic pain and for this reason, many efforts have been spent in this direction [42].

Even later than Nav1.3, the first case of Nav1.6-related epilepsy was diagnosed in 2012 [43]. In the last decades, many mutations of the SCN8 gene have been associated with EIEE, with benign familial infantile seizures-5 (BFIS5) and with several other forms showing a wide spectrum of severity, with symptoms ranging from cognitive and motor regression to cortical blindness [44]. The high number of new variants diagnosed has enabled the identification of several mutational hotspots; interestingly, a mutation localized in the untranslated region of *SCN8A* has been reported that produces a pathogenic variant with a mechanism that could interfere with transcription [45]. Nav1.6 is one of the most common sodium channels in CNS but is also expressed in heart muscle and this can explain the correlation with the increased risk of sudden unexpected death (SUDEP) observed in Na1.6-related epileptic patients [46]. 

As a general rule, it is established that epileptogenic GoF or LoF variants of the Nav CNS isoforms shift in the opposite direction of neuronal excitability and firing, differently affecting neural network activity, depending on whether the channel is prevalent in excitatory or inhibitory neurons. This implies that different approaches and molecular targets will be reasonably required to optimize the pharmacological treatments. Within this topic, an additional degree of complexity is added by the fact that patients with the same mutation may respond differently to the same therapeutic approach. Moreover, about 30% of patients are resistant to current antiepileptic drugs [47].

When planning to modulate Nav channel malfunctions using drugs, the high degree of conservation among Nav isoforms has to be taken into account: for instance, reducing the kinetics of Nav1.2 may result in a reduction of the cardiac Nav1.5. Importantly, it has to be considered that, because of the prevalence of Nav1.1 in regulating the excitability of GABAergic interneurons, prescription of antiepileptic drugs which non-selectively block Na channels is not recommended in Nav1.1-related epilepsies because this could worsen the crisis provoked by the decrease of inhibitory activity; conversely the best approach to design a drug based therapy could imply an enhancement GABA production or stability. Additionally, the use of antisense oligonucleotide (ASO) could be a promising strategy to treat haploinsufficiency [48].

Many treatments to fight epileptic seizures and epilepsy in general have been developed and applied over time and many efforts are still currently spent to exploit new therapeutic strategies. However, going into more detail on the pharmacology of epilepsies caused by Nav channels is not within the scope of this review; regarding this subject, it is possible to consult more exhaustive reviews [49,50].

## 4. VGSC in Intellectual Disability

As discussed above, mutations in all CNS-expressed sodium channel genes (*SCN1A*, *SCN2A*, *SCN3A* and *SCN8A*) cause various forms of epileptic phenotypes, and in the case of *SCN1A* also familial hemiplegic migraine, symptoms that by themselves not necessarily imply the presence of neurodevelopmental problems, autism or neurodegenerative phenotypes (Table 1). However, it is becoming increasingly clear that such phenotypes are often associated with variants of these sodium channel genes, with large differences among the different genes affected.

Rather severe forms of neurodevelopmental disorders, often associated with brain malformations, are found in patients carrying *SCN3A* variants [74]. Affected individuals frequently have developmental and epileptic encephalopathy (DEE) and all patients show some degree of early childhood developmental delay. As a general rule, variants cause a gain of function of the ion channel similar to GoF *SCN1A* variants that lead to FHM3 [35], i.e., a defective inactivation process with increased persistent currents [60]. The severity of the disease is likely related to the fact that *SCN3A* is prominently expressed during fetal development, such that its over-activity somehow results in a compromised development of the brain [74].

Variants of *SCN2A* are frequently associated with autism spectrum disorder and intellectual disability, in addition to infantile seizures [12,75,76,77]. Nav1.2 is widely expressed in the CNS, often co-localizing with Nav1.6, predominantly in glutamatergic excitatory neurons [78,79]. Surprisingly Nav1.2 GoF variants are mostly implicated in benign infantile-onset seizures (BIS) or infantile epileptic encephalopathy followed by developmental delay (IEE), while loss of function variants (for example truncations) cause autism spectrum disorder/intellectual disability with or without childhood-onset seizures [12]. Children with IEE can show microcephaly and cerebral and/or cerebellar atrophy. Mutational hotspots include the S4–S5 segment and the pore loop. Some variants, like K1422E alter ion selectivity and fall out of the usual loss- versus gain-of –function classification [78].

Similarly to *SCN2A*, variants of *SCN8A* (encoding Nav1.6) are often associated with developmental impairment and regression [43] in agreement with the overlapping function of these two genes. The first patient discovered with this disease carried the N1768D variant of a highly conserved asparagine [80]. In heterologous expression systems, the variant led to a dramatic increase in persistent currents and incomplete channel inactivation [80]. Similar to *SCN2A*, both GoF as well as LoF are associated with disease but with different outcomes regarding neurodevelopmental phenotypes. GoF variants are associated with seizures and significant developmental impairment and intellectual disability, while LoF variants are not necessarily associated with seizures [43]. 

*SCN1A*, the Na^+^ channel gene with the largest number of epilepsy-associated variants (mostly Dravet syndrome caused by LoF), is less implicated in neurodevelopmental clinical phenotypes than the other Na^+^ channel genes [54]. Beyond epilepsy, patients can present with autism spectrum disorder and SUDEP. Nevertheless, a few rare variants, like the recurrent T226M, can cause severe epilepsy together with profound developmental impairment [81]. In heterologous expression systems, that variant exhibited hyperpolarizing shifts of both activation as well as inactivation and enhanced fast inactivation [82].

In general, the cellular bases underlying neurodevelopmental phenotypes and possible structural brain anomalies caused by Na^+^ channel gene variants are largely unknown. Maturation of the brain, necessitating specific electrical activity for proper neuronal differentiation and migration, synapse formation, axonal sprouting etc. [83] critically depends on the correct functioning of voltage-gated sodium channels, in agreement with the rather severe phenotypes associated with *SCN3A* variants. The fact that *SCN1A* variants generally cause less severe developmental phenotypes is possibly related to its later upregulation after birth. In addition to defective brain maturation, which is continuing during infancy and childhood, neural network-independent mechanisms, like direct cytotoxicity due for example to Na^+^ overloading could play an additional role [84].

## 5. VGSC in Neurodegenerative Diseases

### 5.1. Alzheimer’s Disease

Alzheimer’s disease (AD) is a heterogeneous neurodegenerative disorder with irreversible progression, characterized by the progressive loss of synapses and neurons and by the formation of amyloid plaques in the brain. Clinically, it is characterized by loss of memory, followed by deterioration of all mental functions, neuronal degeneration of both cerebral and limbic cortices, reactive gliosis and deposition in the brain parenchyma of amyloid aggregates (or plaques) closely associated with dystrophic neurons. Activated phagocytic microglia, and intraneuronal aggregates of disrupted microtubules, known as “neurofibrillary tangles” are also detectable [85]. The molecular mechanisms underlying the development of AD are not well known so far and also the physiological functions of the crucial proteins the amyloid precursor protein (APP) and the presenilins 1 and 2 (PS1 and PS2) are unclear. The toxic extracellular amyloid oligomers detectable in AD plaques are composed of amyloid-Aß peptides (Aß) derived from the sequential proteolytic cleavage of APP by the ß-secretase BACE1 and of the “ß-secretase-complex” in which PS1 has a regulatory role. Mutations of the *APP* gene are responsible for AD as well. In addition, intracellular neurofibrillary tangles, composed of filaments of hyper-phosphorylated Tau protein, are neuropathological hallmarks of AD. Furthermore, presenilins, which are part of the molecular machinery that processes APP, when mutated are responsible for most of the cases of familial AD; more than 70 different mutations in presenilin 1 (PS1) have been associated with inherited early onset Alzheimer’s disease [86]. The phenotypical heterogeneity among patients, and even among familial patients with the same genetic mutation, implies that other proteins might have a role in regulating the onset and severity of the neurodegeneration. Recent findings suggest that APP and PSs are the center of a complex network of interactions with many different intracellular adaptors, but the role of these proteins in the physiology or pathology is still unknown [87]. APP contains a YENPTY motif that has been previously described as an internalization motif, which now has been recognized to be involved as a key player in the regulation of multiple interactions with intracellular proteins [88]. The significance of the motif, which is typical of the receptor (TKR) and non-receptor tyrosine kinases (TK), in amyloid formation and in general for AD development is under investigation. Furthermore, the cytoplasmic tail of APP undergoes post-translational modifications; in particular, one of the mechanisms which may regulate APP cleavage and protein–protein interactions is linked to the occurrence of phosphorylation at Ser, Thr and Tyr residues. For example, Thr 668 can be phosphorylated by c-Jun N-terminal kinase-3 [89]. Thus APP is a tightly regulated protein, post-translationally modified by kinases. The pathophysiological significance of APP phosphorylation is unclear and there are even contrasting opinions about the effective influence of such post-translational modifications on APP cleavage, amyloid formation and AD development. Indeed in this context, it has to be underlined that APP and APP-related proteins, APLP1 and APLP2, can interact with several proteins such as X11 and Fe65 [90], c-Abl [91], mDab [92], JIP-1 [93] independently of the phosphorylation of the tyrosine residues of the YENPTY motif.

Interestingly, several studies suggest that the physical interaction of APP and its related proteins, Aß oligomers and BACE1 enzyme with various isoforms of Nav could interfere with neural physiological processes and be involved in the development of the AD.

#### 5.1.1. Alzheimer Disease and Nav Involvement: APP Phosphorylation

Even if a causative dependence of AD from VGSC mutations has not been assessed so far, it has been recently hypothesized that several isoforms may be modulated from APP. In a paper published in 2015, Liu and collaborators demonstrated that in murine cortical neurons, APP co-localizes and interacts with Nav1.6 and that knocking down APP provokes a decrease in Nav1.6 cell surface expression and function [94]. Conversely, APP-induced increases of Nav1.6 cell surface expression have been shown to be dependent on Go protein, the most abundant G protein in the CNS, being enhanced by a constitutively-active mutant Go protein and blocked by a dominant negative mutant Go protein. Interestingly, Nav1.6 sodium channel surface expression was shown to be increased by T668E and decreased by T668A mutations of APP, mimicking and preventing Thr-668 phosphorylation, respectively. In agreement phosphorylation of APP at Thr-668 enhanced its interaction with Nav1.6. Furthermore, APP regulates JNK activity in a Go protein-dependent manner and JNK, in turn, phosphorylates APP. Therefore, APP enhances Nav1.6 sodium channel cell surface expression through a Go-coupled JNK pathway [94]. The interaction between APP and Nav1.6 sodium channel was further studied by Shao li and colleagues. From studies in APP knockout mice they observed that APP molecules aggregated at nodes of Ranvier (NORs) in CNS myelinated axons and interacted with Nav1.6 and described a reduction of sodium current density in hippocampal neurons as well. Coexpression of APP or its intracellular domains (AICD) with Nav1.6 in *Xenopus laevis* oocytes resulted in an increase of peak sodium currents, which also in this case was enhanced by constitutively-active Go mutant and blocked by a Go dominant negative mutant. Similarly to the results of Liu and colleagues, Nav1.6 current was increased by APP mutation T668E and decreased by T668A. Accordingly, the cell surface expression of Nav1.6 sodium channels in the white matter of the spinal cord and the spinal conduction velocity was decreased in APP/JNK3/knockout mice. Thus also in this study, the conclusion was that APP modulates Nav1.6 sodium channels through a Go-coupled JNK pathway and that this modulation is dependent on the phosphorylation of the Thr668 residue of APP [67].

#### 5.1.2. Alzheimer’s Disease and Nav Involvement: Aß Oligomers

The correlation among AD pathophysiology, seizures and increased neuronal excitability was established by Busche and colleagues examining an AD mouse model. Indeed in the CA1 hippocampal region of young APP/PS1 mice, they observed an increased number of hyperactive neurons associated with the high level of Aβ oligomers produced, and therefore speculated that soluble Aβ oligomers might directly induce neuronal hyperactivity [95]. Several lines of evidence indicated that amyloid-β1–42 (Aβ1–42) induced neuronal hyperactivity may give rise to cognitive deficits and memory dysfunction in AD. Indeed recent studies on primary hippocampal neurons of another AD mouse model (Tg2576) exposed to Aβ1–42 oligomers, demonstrated that the overexpression of Nav1.6 contributes to membrane depolarization and to the increase of spike frequency, thereby resulting in neuronal hyperexcitability. These findings identify the Nav1.6 channel as a determinant of hippocampal neuronal hyperexcitability induced by Aβ1–42 oligomers [96].

#### 5.1.3. Alzheimer’s Disease and Nav Involvement: BACE1

Another relevant molecule for the sequential proteolytic cleavage of APP and plaques formation is the ß-secretase BACE1. Some authors describe a correlation between BACE1 and sodium channel expression. A recent study performed by De-Juan Yuan and colleagues on WT mice and on the APP/PS1 AD mouse model has shown that Nav1.6 is overexpressed in old AD mice. The high expression of Nav1.6 in APP/PS1 mice enhances BACE1 transcription through activation of the NFAT1 factor regulated by the Na(+)/Ca(2+)exchanger (NCX). Interestingly, the authors demonstrated that knocking down Nav1.6 with a bilateral injection of adeno-associated viruses (serotype 8, AAV8) encoding shRNA of Nav1.6 in the hippocampus significantly reduced the density of Aβ plaques through the suppression of the β-secretase-mediated cleavage of APP. As a consequence, the cognitive deficit of the mice and the neural network hyper excitability were both remarkably reduced [68]. The authors went further in defining the molecular mechanisms underlying the role of Nav1.6 in AD pathogenesis. Treating Nav1.6 overexpressing HEK cells with TTX, an unspecific blocker of CNS VGSC, they observed a remarkable reduction of BACE1 expression; the same reduction was less evident when Nav1.6 channel was knocked down by shRNA transfection and TTX was applied, unveiling a molecular mechanism dependent on Na ion flux and not only the presence of the channel itself. Thus for the first time, Nav1.6 has been indicated as a new target to be considered to slow down AD evolution. 

Other authors suggest that Nav1.1 and 1.2 might also be involved to some extent in AD; indeed the results of their investigations indicated a reduction of Nav1.1, 1.2 and Nav1.6 α-subunits protein in primary neurons in a culture of wild type BACE1-null mice. They propose an underlying mechanism involving BACE1 activity regulating mRNA levels of the Nav1.1 α-subunit through the cleavage of the Navβ2 subunit expressed on the surface. Interestingly in the hippocampus of the same murine model, Nav1.1 expression appeared significantly reduced, while Nav1.2, perhaps as a compensatory mechanism, was remarkably increased. Thus endogenous BACE1 activity seems to regulate total and surface levels of VGSC in mouse brains [53].

### 5.2. Parkinson’s Disease

Parkinson’s disease (PD) is a neurodegenerative disorder characterized by motor disabilities that affects predominantly the dopaminergic neurons of the substantia nigra causing a decrease in dopamine levels in the striatum [97]. The main symptoms are bradykinesia, akinesia, muscle rigidity, postural instability, stiffness and resting tremor which may be due to the high levels of synchronous oscillations in the basal ganglia neurons [98,99]. The causes of PD are unknown, although it is speculated that there may be a contribution from genetic and environmental factors [100]. PD pathogenesis has been associated with a number of factors, including impairments linked to intracellular Ca^2+^ excess, mitochondrial malfunction, oxidative or metabolic stress, and, in particular, a small number of neurotoxins that render neuronal cells more susceptible to death. VGSCs have an important role in the abnormal electrical activity of neurons in the globus pallidus and the subthalamic nucleus in PD [101] and are involved in cognitive impairments. By using the rat PD model infused with 6-OHDA (6-hyroxydopamine), Wang and colleagues showed that the expression of Nav1.1, 1.3 and 1.6 in the hippocampus was dynamically increased at different time points after dopamine depletion [61]. In contrast, treating rats with phenytoin, a sodium channel blocker that slows down the recovery from inactivation [102], remarkably improved cognitive impairments. In MPTP (1-metyl-4-fenyl-1,2,3,6-tetrahydropyridin)-treated PD mice it was found that Nav1.1 expression was increased in the external globus pallidus [103]. Globus pallidus is a central nucleus of the basal ganglia; it receives the majority of the inhibitory GABAergic inputs from the striatum and plays a key role in the propagation of synchronized oscillatory activity of basal ganglia [99]. In particular, the increase of Nav1.1 expression in MPTP-treated mice was evident in parvalbumin (PV) positive GABAergic interneurons that exhibit fast-firing spontaneous activity, and exert their inhibitory control on the activity of innervated neurons in the subthalamic nucleus, substantia nigra and in the striatum [56]. In these cells, Nav1.1 is a determinant for the maintenance of sustained fast spiking more than for its onset [104,105]. Additionally, in this study phenytoin was used to test the effectiveness of blocking VGSCs in reducing PD symptoms. Indeed both motor disability and high synchronous oscillations were reduced in MPTP-treated mice, thus confirming the potential therapeutic role of this compound in PD. Even if the role of GABAergic transmission in PD is still unknown, it is possible to speculate that the observed effect of phenytoin could result from blocking the increased activity of Nav1.1 in the globus pallidus thus restoring the physiological GABAergic activity. Probably, the upregulation of Nav1.1 expression in globus pallidus may be a compensatory molecular mechanism aimed to enhance inhibitory response in the basal ganglia and counteract the abnormal neural activity of PD animals. Nav1.3 seems to be involved in PD as well. This VGSC isoform generally is robustly expressed during the fetal period and is downregulated after birth. By using the rat PD model infused with 6-OHDA, it has been demonstrated that 49 days after infusion Nav1.3 is re-expressed in dopaminergic neurons of the substantia nigra [61]. Additionally, in this case the authors suggest that the re-expression of Nav1.3 could be a compensatory mechanism for the degeneration of dopaminergic neurons caused by PD progression. Current therapies treat only PD symptoms, but several investigations have also been carried out in order to find putative neuroprotective drugs for dopaminergic neurons. In particular, Sadeghian and colleagues have examined the effects of safinamide on microglial activation and dopaminergic neurons degeneration in a rat model of PD in vivo. In the PD rat model, safinamide reduced the number of activated microglial cells and increased survival of dopaminergic neurons [106]. Safinamide is a sodium and calcium channels modulator and inhibits glutamate release induced by abnormal neuronal activity, promoting its neuroprotective effect [107]. Specifically safinamide interacts with the inactivated state of the VGSCs, keeping most of the channels in the inactive state and preventing their activation. This effect induces a selective depression of the pathological high-frequency firing, leaving physiologic activity unaffected and thus avoiding CNS depressant effects [108].

### 5.3. Amyotrophic Lateral Sclerosis (ALS)

Amyotrophic Lateral Sclerosis (ALS) is an unknown etiology disease, caused by the progressive neurodegeneration of motor neurons [109]. The degenerative process induces a progressive atrophy of the neuromuscular system which causes death from paralysis 3–5 years after the onset of the disease [110]. At the moment there is no effective therapy for ALS on slowing down or arresting the neurodegenerative process [111]. To date, there are two main drugs for the treatment of ALS aimed to prolong the patient’s life expectancy: riluzole [109,112] and edaravone [112,113]. Although the mechanisms by which these drugs exert their effects are not well known, various hypotheses have been formulated. Riluzole is believed to modulate the release of glutamate [114,115] and sodium channel activity. In particular, this drug is able to down-regulate neuronal firing and inhibit the persistent current of VGSC [116,117]. Persistent current (see Figure 1) is caused by a particular kinetics of VGSC characterized by a rapid activation followed by a subsequent slow inactivation which maintains the channel in the activated state for hundreds of milliseconds [118]. It is generally more evident in various pathological conditions, when the ionic environment is altered or when a mutation modifies functional properties of VGSCs and contributes to maintaining the neuronal membrane potential near the threshold value triggering spontaneous action potentials [119]. Vucic and Kiernan using the transcranial magnetic stimulation technique on ALS patients have demonstrated that cortical excitability is abnormally increased in an early state of the disease [120]. Similarly experiments performed on ASL animal models have confirmed that a neuronal hyperexcitability of the motor cortex activating the glutamate excitotoxic cascade via trans-synaptic mechanism is at the route of the neurodegenerative process of the motor neuron [121,122]. 

Although the molecular mechanisms underlying ALS are still not well understood, several pieces of evidence indicate that Nav1.6 channels could be a potential therapeutic target. Using G93A mice, Saba and collaborators showed that the expression levels of the Nav1.6 channels during ASL progression are modified in the primary motor cortex but not in other cortical areas with consequent alteration of the excitability and of persistent current of this neural district [69].

While it is assumed that Nav1.6 dysfunction may be linked to ALS, evidence has recently been provided of the existence of sporadic ALS forms caused by heterozygous point mutations in the *SCN4A* gene that precede the development of the disease. In this context, two mutations, Arg672His and Ser1159Pro, which have opposite effects on neuromuscular excitability have been identified in two different patients. In both cases, the authors hypothesize that the abnormal Nav1.4 channels predisposed to depolarization-induced cellular excitotoxicity, leading to the development of ALS [62]. Whole genome sequencing analysis of ALS patients identified the presence of missense mutations in the *SCN7A* gene, which codes for NaX, a type II sodium channel sensitive to the extracellular [Na^+^] [66]. Mutations in this channel result in a loss of function phenotype which provokes a dysregulation of sodium homeostasis and neuronal hyperexcitability. Overall, the findings described above support the hypothesis of the role of VGSC dysfunction in ALS development. Gaining more insights into the molecular mechanisms related to VGSC underlying the disease could lead to the identification of new therapeutic targets and even pave the way for personalized gene therapy.

### 5.4. Multiple Sclerosis

Multiple sclerosis (MS) is a multifactorial neurodegenerative disease of the central nervous system whose etiology is still mostly unknown. It is a chronic demyelinating disease characterized by an autoimmune response against the tissues of the central nervous system with lymphocytic and macrophage infiltration [123]. The pathological hallmarks of MS are demyelinated plaques in the CNS with inflammation, gliosis, and neurodegeneration [124]. At the beginning of the disease, the lymphocyte infiltration that triggers the axonal and myelin damage can be recovered. Later the inflammatory episodes occur repeatedly and microglia activation causes extensive and chronic neurodegeneration leading to disability. 

An experimental autoimmune encephalomyelitis (EAE) mouse model is used to study MS. In this murine model, T cells infiltrate the CNS, initiate demyelination and cause loss of axons [125]. VGSCs have an important role in axonal loss in MS. It has been shown that in demyelinated axons there is a particular distribution of sodium channels, with Nav1.2 and Nav1.6 present in the plaques together with the Na^+^/Ca^2+^ exchanger whereas in non-demyelinated control axons Nav1.6 is located only in Ranvier nodes [72]. High sodium flux along Nav1.6 reverses the Na^+^/Ca^2+^ exchanger and increases axonal calcium finally leading to axonal damage through the activation Ca^2+^ dependent proteases [72]. Upregulation of Nav1.8 detected in cerebellar Purkinje neurons of MS patients and in the experimental EAE mouse model appears to be a determinant for the cerebellar dysfunction observed in this disease [64,73]. Indeed the administration of a selective Nav1.8 blocker in the cerebrospinal fluid of EAE mice partially improved symptomatology [126]. Overexpression of Nav1.5 has been detected in astrocytes of post-mortem MS brain tissue. It has been suggested that its upregulation is necessary to restore physiological ATPase-dependent Na^+^/K^+^ homeostasis in damaged neural areas [64]. Experiments performed on an in vitro model of glial injury [127] have assessed that Nav1.5 in non-excitable cells plays the main role of reversing the NCX function. It has been proposed that the application of sodium channel blockers could attenuate the inflammatory effects provoked by microglia injury and activation such as phagocytosis, the release of cytokines interleukin-1 and tumor necrosis factor-α [128]. In vitro studies have shown that Nav1.5 is present in the membrane of maturing endosomes of macrophages suggesting a possible role of the channel in the phagocytic pathway of myelin degradation within MS lesions [65].

## 6. Conclusions

The purpose of this review was to collect and summarize the main information currently available in the scientific literature on the multifaceted role of Nav channels on pathologies affecting the CNS, whether neurodegenerative or not. We expect that having all the data available in the literature on such a complex subject grouped together can effectively support future scientific activity on this topic.

A picture emerged in which malfunctions of the same brain Nav isoform can lead to very different neural pathologies; for instance, Nav1.1 mutations may cause several forms of epilepsy or genetic migraine, but the channel is suspected to also be involved in autism spectrum disorders; similarly, Nav1.2 is correlated with epilepsy and with several forms of autism; finally Nav1.6 when mutated gives rise to epilepsy, whereas when its expression is impaired is correlated with developmental regression or with the most common neurodegenerative diseases such as Alzheimer’s and Parkinson’s. 

Even if some of the pathologies described are not directly categorized as neurodegenerative, nevertheless some of their manifestations can produce neurodegeneration as well. Epileptic crises with prolonged seizures are clearly capable of injuring the brain, while brief and isolated seizures are likely to cause negative changes in brain function and possibly the loss of specific brain cells.

In order to better dissect and elucidate the molecular basis of Nav-correlated brain pathologies a tight collaboration between clinicians and researchers is essential to obtain new experimental models and techniques. 

Many efforts have been spent until now to find new treatments to limit neuronal damage once the pathology has manifested. 

Currently, therapies that intervene by blocking the degenerative process, reducing neuronal loss and restoring nerve transmission, are not available. There is numerous clinical evidence which suggests that antioxidant substances, for example, are useful both in preventing and modifying the course of neurodegenerative diseases.

Further studies on neuroprotection as a therapeutic intervention aimed at slowing or even halting the progression of neurodegeneration would be desirable.

## Figures and Tables

**Table 1:** List of the main neurological and neurodegenerative diseases correlated with Nav channel dysfunctions.

Nav Isoform	Gene	Neurological Disorder	References	Neurodegenerative Disorder	References
Nav1.1	*SCN1A*				
		Dravet Syndrome	[51,52]	Alzheimer’s Disease	[53]
		GEFS+ (genetic epilepsy with febrile seizures plus)	[52,54,55]	Parkinson’s Disease	[56]
		Epilepsy of infancy with migrating focal seizures	[52,54]		
		Myoclonic-atonic epilepsy	[52,54]		
		Familial hemiplegic migraine	[32]		
Nav1.2	*SCN2A*				
		Developmental and epileptic encephalopathy (DEE).	[52,57]	Alzheimer’s disease	[53]
		Benign Familial Neonatal-Infantile Seizures (BFNIS)	[52,57]		
		West syndrome	[38,58]		
		Epilepsy of infancy with migrating focal seizures (EIMFS)	[52,54]		
		Autism Syndrome Disorder (ASD)	[58]		
		Intellectual Disability	[58]		
		Episodic ataxia	[59]		
Nav1.3	*SCN3A*				
		Developmental and Epileptic Encephalopathy (DEE)	[60]	Parkinson’s Disease	[61]
		Polymicrogyria	[60]		
		Intellectual Disability	[60]		
Nav1.4	*SCN4A*				
				Amyotrophic Lateral Sclerosis	[62]
				Huntington’s Disease	[63]
Nav1.5	*SCN5A*				
				Multiple Sclerosis	[64,65]
NaX	*SCN7A*				
				Amyotrophic Lateral Sclerosis	[66]
Nav1.6	*SCN8A*				
		Developmental and epileptic encephalopathy (DEE).	[52]	Alzheimer Disease	[67,68]
		Autism Syndrome Disorder (ASD)	[52]	Amyotrophic Lateral Sclerosis	[69]
		Intellectual Disability	[70,71]	Multiple Sclerosis	[72]
Nav1.8	*SCN10A*				
				Multiple Sclerosis	[64,73]

## Data Availability

No new data were created or analyzed in this study. Data sharing is not applicable to this article.
